# *Citrus ichangensis* Peel Extract Exhibits Anti-Metabolic Disorder Effects by the Inhibition of PPAR**γ** and LXR Signaling in High-Fat Diet-Induced C57BL/6 Mouse

**DOI:** 10.1155/2012/678592

**Published:** 2012-12-19

**Authors:** Xiaobo Ding, Shengjie Fan, Yan Lu, Yu Zhang, Ming Gu, Lu Zhang, Gaigai Liu, Lu Guo, Dong Jiang, Xiong Lu, Yiming Li, Zhiqin Zhou, Cheng Huang

**Affiliations:** ^1^College of Horticulture and Landscape Architecture, Southwest University, Chongqing 400716, China; ^2^Key Laboratory of Horticulture Science for Southern Mountainous Regions, Ministry of Education, Chongqing 400715, China; ^3^School of Pharmacy, Shanghai University of Traditional Chinese Medicine, 1200 Cailun Road, Shanghai 201203, China; ^4^Citrus Research Institute, Chinese Academy of Agricultural Sciences, Chongqing 400712, China; ^5^Scientific Experimental Center, Shanghai University of Traditional Chinese Medicine, 1200 Cailun Road, Shanghai 201203, China

## Abstract

Obesity is a common nutritional disorder associated with type 2 diabetes, cardiovascular diseases, dyslipidemia, and certain cancers. In this study, we investigated the effects of *Citrus ichangensis* peel extract (CIE) in high-fat (HF) diet-induced obesity mice. Female C57BL/6 mice were fed a chow diet or an HF diet alone or supplemented with 1% w/w CIE for 8 weeks. We found that CIE treatment could lower blood glucose level and improve glucose tolerance. In the HF+CIE group, body weight gain, serum total cholesterol (TC) and low-density lipoprotein cholesterol (LDL-c) levels, and liver triglyceride (TG) and TC concentrations were significantly (*P* < 0.05) decreased relative to those in the HF group. To elucidate the mechanism of CIE on the metabolism of glucose and lipid, related genes expression in liver were examined. In liver tissue, CIE significantly decreased the mRNA expression levels of peroxisome proliferator-activated receptor **γ** (PPAR**γ**) and its target genes, such as fatty acid synthase (FAS) and acyl-CoA oxidase (ACO). Moreover, CIE also decreased the expression of liver X receptor (LXR) **α** and **β** which are involved in lipid and glucose metabolism. These results suggest that CIE administration could alleviate obesity and related metabolic disorders in HF diet-induced obesity mice through the inhibition of PPAR**γ** and LXR signaling.

## 1. Introduction

According to World Health Organization (WHO) estimates, there are 1.4 billion overweight adults worldwide and more than 500 million of these are obese [1]. Obesity is one of the most notorious symptoms of metabolic disorders throughout the world. It is considered to be a major risk factor for various chronic diseases, including type 2 diabetes, major cardiovascular diseases, hypertension, dyslipidemia, and certain cancers [2]. At present, only orlistat can be used for long-term weight reduction. However, these drugs are confounded by diminishing response in long-term treatment because of side effects and limited efficacies [3, 4]. Nutritional components may play a prominent role in the prevention and treatment of obesity and related metabolic disorders. Recently, there have been increasing efforts in research for new health-enhancing foods from natural products, and these findings also suggest that nutritional intervention could be an effective and promising strategy to inhibit obesity and obesity-related metabolic diseases [5, 6].

Citrus is one of the most important fruits in the world and is a rich source of nutrients and bioactive compounds. Citrus fruits not only provide ample vitamins, minerals, dietary fibers, and pectins but also provide an abundant of bioactive compounds, including flavonoids, coumarins, limonoids, and carotenoids. Currently, the study of bioactive compounds is one of the most active fields of food and medical science. Many epidemiological and experimental studies have provided convincing evidence that the intake of citrus fruits is beneficial to health [7–9]. Numerous prevention and treatment properties have been attributed to citrus fruits, like antioxidant, antiinflammatory, antitumor, anticardiovascular, and antiobesity properties [10–14]. Citrus fruits are usually consumed as fresh product or juice with peels and seeds discarded. Regretfully, these wastes are abundant sources of natural bioactive compounds [15]. In China, citrus peels like *chenpi* (dried peels of mature *C. reticulate*) or *qingpi* (dried peels of immature tangerine (*C. reticulate*)) have been commonly used in Chinese medicine for the treatment of a number of diseases, such as indigestion, bronchial asthma, vomiting, cough, skin inflammation, and muscle pain [16, 17]. Moreover, citrus peels have been extensively consumed as baked products, culinary seasonings, preserves, and food supplements in China for centuries. Recently, the prevention and treatment of obesity and obesity-related metabolic diseases of citrus peels have received increasing attention. Jung et al. found that *Citrus unshiu* peel extract inhibited lipid and triglyceride accumulation in 3T3-L1 adipocytes [18]. The extract from *Citrus unshiu* Mark induced the lipolysis* in vitro *[19]. A study by Bok et al. suggested that citrus peel diet reduced plasma and hepatic cholesterol in rats [20]. It has been reported that the immature *Citrus sunki* peel extract had an antiobesity effect by elevated *β*-oxidation and lipolysis in the adipose tissue of HF diet fed mice [21]. In addition, citrus phytochemicals, such as flavonoids, nomilin, synephrine, and auraptene, have exhibited antiobesity effects by increasing energy expenditure, improving metabolism, and enhancing lipolysis [22–25]. 

Emerging evidence suggests that PPARs are the targets of many citrus-derived flavonoids [26]. PPAR is the nuclear receptor transcription factor that is known to regulate carbohydrate and lipid metabolism in various tissues and cells [27]. The PPAR family includes three isoforms: PPAR*α*, PPAR*γ*, and PPAR*δ*/*β*. PPAR*γ* is an important regulator of adipocyte differentiation, lipogenesis, and glucose metabolism [28, 29]. Citrus flavonoids have been shown to inhibit adipogenesis and to decrease adiposity which can be explained in part by regulating the PPAR expression levels both *in vivo* and *in vitro *[30, 31]. It has been previously shown that citrus polymethoxylated flavones improve lipid and glucose homeostasis and restore insulin sensitivity through regulating the expression of PPAR*α* and PPAR*γ* [32, 33]. A recent study has suggested that *Citrus aurantium* flavonoids suppressed adipogenesis by inhibiting the expression of PPAR*γ* in 3T3-L1 cells [31]. Studies have also identified that LXR is a target for metabolic diseases [34]. The citrus component naringin decreases serum lipid through the increase of PPAR*γ* expression and inhibition of LXR expression in the liver of type 2 diabetic rats [34]. The grapefruit flavonoid naringenin has been reported to be an agonist of PPAR*α* and PPAR*γ*, and a partial agonist of LXR*α* [26].

Although citrus fruits are widely used in the pharmaceutical and food industries, researches on the functions of endemic citrus species remain insufficient. China has much abundant germplasm resources of the citrus fruits, but there is underutilization of these citrus resources. *Citrus ichangensis *Swingle is a unique citrus species grown in China and is known by its unusual hardiness and contains a wide range of bioactive compounds [35]. In horticulture, *Citrus ichangensis* was mainly used as rootstock of cultivated citrus due to its stress resistance and the fruit of *Citrus ichangensis* has been used in traditional Chinese medicine for a long history. It has been shown that *Citrus ichangensis *contains the complex pattern of flavones and a large amount of nonbitter deacetylnomilin [36]. Here, we investigate whether the long-term administration of *Citrus ichangensis* peel extract (CIE) would have beneficial effects on the prevention and treatment of obesity and its related metabolic diseases. In the present study, CIE was tested for body weight gain, lipid accumulation, and gene expression involved in glucose and lipid metabolism in HF diet-induced C57BL/6 mice. 

## 2. Material and Methods

### 2.1. Preparation of *Citrus ichangensis *Peel Extract (CIE)

*Citrus ichangensis* Swingle was provided by the Citrus Research Institute, Chinese Academy of Agricultural Sciences, Chongqing China. Samples were prepared by adding 4 L of 95% ethanol to one kilogram of fresh citrus peel, extracting at 85°C for 2 h, cooling, and filtering the solution. The filtered solution was concentrated at 40°C with a rotary evaporator under reduced pressure, freeze-dried to a powder, and stored at −20°C until use. The frozen dried powder of CIE was added to the HF diet for the experiment.

### 2.2. HPLC Analysis

To determine the flavonoids content of CIE, high-performance liquid chromatography (HPLC) analysis was performed on an Agilent 1200 liquid chromatograph system. The flavonoid compounds were monitored at 280 nm using a Discovery C18 HPLC Column (250 × 4.6 mm, 5 *μ*m). The column was operated at 30°C, and the injection volume was 10 *μ*L. The mobile phase consisted of 100% acetonitrile (A) and water containing 0.5% acetic acid (B) at a flow rate of 1.0 mL/min. The gradient profile was as follows: 0–12 min, 85–75% B; 12–17 min, 75% B; 17–20 min, 75–50% B; 20–30 min, 50–25% B; 30–35 min, 25–5% B; and 35–40 min, back to 85% B.

### 2.3. Animals and Diets

The animal study protocols were approved by the Shanghai University of Traditional Chinese Medicine. Four-week-old female C57BL/6 mice were purchased from the SLAC Laboratory (Shanghai, China). Mice were kept under 22-23°C on a 12 h light/dark cycle. After a one-week adaptation period, C57BL/6 mice were randomly separated into three groups (*n* = 7) and were fed a chow diet (10% of calories derived from fat, Research Diets; D12450B), or an HF diet (60% of calories derived from fat, New Brunswick, NJ, Research Diets; D12492), either alone or supplemented with 1% CIE (HF+CIE) diet for 8 weeks. Food intake and body weight were measured every other day. The mice were given free access to food and water.

### 2.4. Intraperitoneal Glucose Tolerance Test

For intraperitoneal glucose tolerance test (ipGTT), all the mice were fasted for 12 h and a basal blood glucose levels (0 min) were determined from the tail vein. The mice were then intraperitoneally injected with glucose (1 g/kg body weight), and additional blood glucose levels were measured at 15, 30, 60, and 90 min. 

### 2.5. Serum Chemistry Analysis

After overnight fasting, all mice were anesthetized with urethane before collecting blood samples for analysis. Blood samples were drawn from the heart into a vacuum tube and allowed to clot at room temperature for 30 min. Serum samples were separated from the blood and the serum triglyceride (TG), total cholesterol (TC), low-density lipoprotein cholesterol (LDL-c), and high-density lipoprotein cholesterol (HDL-c) were analyzed using a Hitachi 7020 Automatic Analyzer. Serum lipid parameters were measured by Clinical Reagents following the manufacturer's instructions.

### 2.6. Liver and Fecal Lipid Content Analysis

The liver and other tissues were rapidly collected at the end of treatment, frozen in liquid nitrogen, and stored at −80°C for further experiments. 50 mg of frozen liver tissue was minced and homogenized in 1 mL of tissue lysis buffer (20 mM Tris·HCl, pH 7.5, 150 mM NaCl, 1% Triton) and mixed with an equal volume of chloroform. The chloroform layer was separated, dried, and resuspended in 100 *μ*L of isopropyl alcohol to measure the lipid levels as described above. Fecal lipids were extracted and measured as described above.

### 2.7. Morphological Analysis of Epididymal WAT

Scanning electron microscopy was used to examine the structure of epididymal white adipose tissue (WAT) by the method developed by Chun et al. [37]. The epididymal fat pads were fixed with 10% neutral formalin and postfixed in 1% osmium tetroxide. Samples were viewed using a Philip XL-30 scanning electron microscope with a magnifying power of ×200.

### 2.8. Quantitative Real-Time RT-PCR

Total RNA from liver tissue was extracted with a spin column (Qiagen, Germany) according to the manufacturer's protocol. The first-strand cDNA was synthesized using the cDNA synthesis kit (Fermentas, Madison, WI, USA). The gene expression levels were analyzed by quantitative real-time RT-PCR conducted using the ABI StepOnePlus real-time PCR system (Applied Biosystems, USA). The primers involved in the experiments were shown in Table 1. The cDNA was denatured at 95°C for 10 min followed by 40 cycles of PCR (95°C, 15 s, 60°C, 60 s). All results were obtained from at least three independent experiments. The expression levels of genes were normalized using *β*-actin as an internal control.

### 2.9. Statistical Analysis

All values are expressed as the mean ± SD unless otherwise indicated. Data analysis was performed by SPSS 12.0 software for Windows statistical program. Statistical analysis was programmed by one-way analysis of variance (ANOVA). Differences were defined as significant when *P* < 0.05.

## 3. Results

### 3.1. Flavonoid Contents in CIE

**Figure 1:**
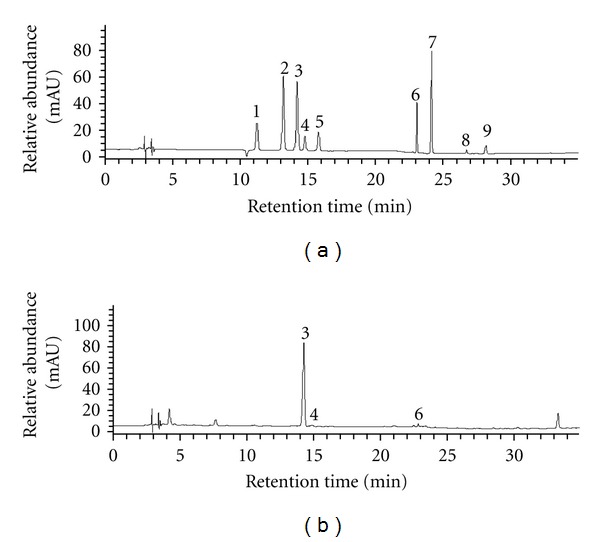
HPLC chromatograms of major flavonoids of citrus peel extract (CIE). (a) (1) Neoeriocitrin; (2) narirutin; (3) naringin; (4) hesperidin; (5) neohesperidin; (6) poncirin; (7) naringenin; (8) nobiletin; (9) tangeretin were analyzed as standards. (b) The major flavonoid components of CIE were determined to be compared to retention time of the chromatogram of standard: (3) naringin; (4) hesperidin; (6) poncirin.

The flavonoid composition of CIE was detected through HPLC analysis.  Figure 1 shows the levels of major citrus fruit flavonoids including neoeriocitrin, narirutin, naringin, hesperidin, neohesperidin, poncirin, naringenin, nobiletin, and tangeretin. The major flavonoids in CIE were naringin (8.12 mg/g), hesperidin (0.84 mg/g), and poncirin (1.33 mg/g). Naringin, the glycoside form of naringenin abundantly found in citrus fruits, possesses a wide range of pharmacological activities including antioxidative stress, anti-inflammatory, and anticancer effects.

### 3.2. CIE Blocks Body Weight Gain C57BL/6 Mouse Induced by HF Diet-Induced

**Figure 2:**
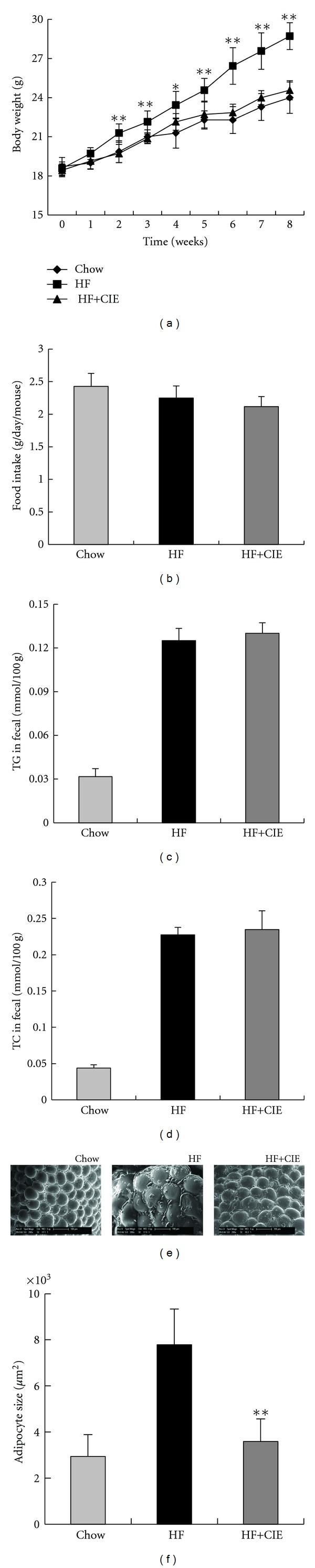
CIE prevents HF diet-induced weight gain. C57BL/6 mice were fed a chow diet (Chow) or an HF diet alone (HF) or supplemented with 1% of CIE (HF+CIE) for 8 weeks. (a) Body weight change; (b) food intake expressed as g/day/mouse; (c) TG concentrations in fecal material; (d) TC concentrations in fecal material;  (e) epididymal WAT morphology are shown at 200x; (f) size of adipocytes. Values are expressed as means ± S.E. (*n* = 7; *P < 0.05, **P < 0.01 versus the HF group).

To test the effects of CIE on the metabolic disorders, we fed the female C57BL/6 mice with a chow diet (Chow), or an HF diet alone or supplemented with 1% CIE (HF+CIE) diet for 8 weeks. The results showed that the mean body weight gain of the HF group was 91.9% more than those in the Chow group after 8 weeks of treatment, indicating the HF diet-induced obesity (Figure 2(a)). The body weight gain induced by HF diet was significantly suppressed by treatment with CIE from 2 weeks into the treatment period to the end of treatment (HF+CIE group). In this study, food intake in the HF+CIE group was roughly equivalent to that in the HF group (Figure 2(b)). As shown in Figures 2(e) and 2(f), the size of epididymal adipocytes was significantly elevated in the HF group compared to the Chow group after 8 weeks. Adipocyte size was markedly decreased in the HF+CIE group compared to the HF group. Furthermore, the concentrations of TG and TC in fecal material were slightly but not significantly higher in the HF+CIE diet-fed mice than the HF diet-fed mice (Figures 2(c) and 2(d)). This indicated that CIE slightly decreased lipid absorption or increased lipid excretion to antagonize diet-induced obesity. These results suggested that CIE could prevent diet-induced obesity independent of food intake inhibition and lipid absorption in the intestine. 

### 3.3. CIE Improves Glucose Tolerance and Attenuates Dyslipidemia

**Figure 3:**
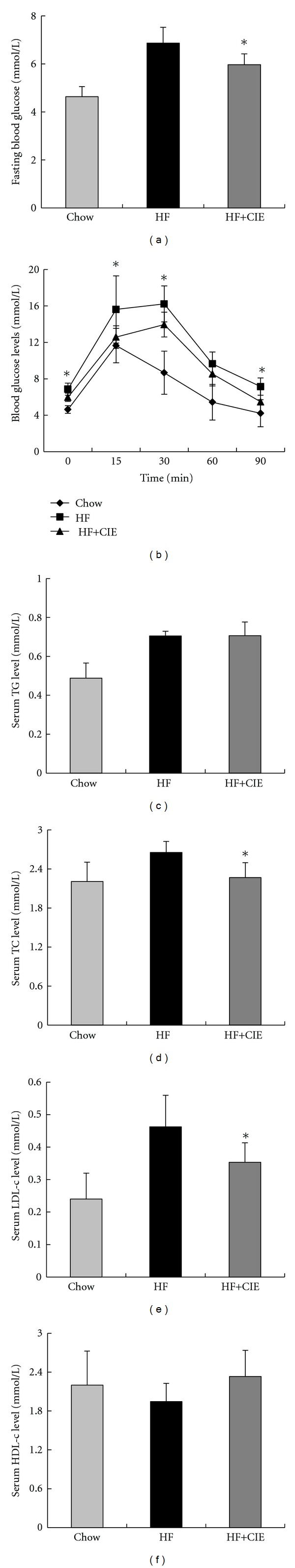
CIE decreases the blood glucose and serum lipid levels in HF diet-fed mice. Mice were fed a chow diet (Chow) or an HF diet alone (HF) or supplemented with 1% of CIE (HF+CIE) for 8 weeks. (a) Fasting blood glucose concentration. The mice were fasted for 12 hours and the tail vein blood was used to test the glucose level. (b) Glucose tolerance test was performed by intraperitoneal injection of glucose (1 g/kg body weight) into mice and blood glucoses were measured at 0, 15, 30, 60, and 90 min. ((c)–(f)) TG, TC, LDL-c and HDL-c concentrations in serum from fasted mice. Values are expressed as means ± S.E. (*n* = 7; *P < 0.05, **P < 0.01 versus the HF group).

To understand the effects of CIE on the metabolic disorders, we analyzed the serum biochemical contents in the mice. HF fed mice showed a significant increase in fasting blood glucose levels compared to the Chow group mice (*P* < 0.05). In contrast, the CIE groups showed a statistically significant (*P* < 0.05) decrease in fasting blood glucose levels compared to the HF group (Figure 3(a)). 

We further tested ipGTT of the mice. As shown in Figure 3(b), the glucose levels were significantly increased in the HF group mice at 15, 30, 60, and 90 min following the injection of glucose, whereas the blood glucose levels in CIE-treated mice significantly decreased at 15, 30, and 90 min compared to the HF group. The total area under the curve of blood glucose levels between 0 to 90 min was 11.6 ± 1.7 mmol/L/min for the HF group and 9.8 ± 1.2 mmol/L/min for the HF+CIE group (*P* < 0.05). The results indicate that CIE treatment improved the glucose tolerance induced by an HF diet in the mouse.

The fasting serum TG, TC, and LDL-c concentrations of HF group were increased by 44.4%, 20.3%, and 91.1%, respectively, compared to the Chow group (Figures 3(c)–2(e)), whereas the level of HDL-c decreased by 8.7% when compared to the Chow group (Figure 3(f)). The levels of TC and LDL-c were markedly reduced by CIE treatment in HF diet-induced mice. However, a slight increase was observed in HDL-c in the HF+CIE groups when compared to the HF group. We did not observe the differences of the TG levels between the HF and HF+CIE groups (Figure 3(f)). These results indicate that CIE is effective in attenuating high-fat diet-induced risk for dyslipidemia *in vivo*.

### 3.4. CIE Prevents Hepatic Lipid Accumulation

**Figure 4:**
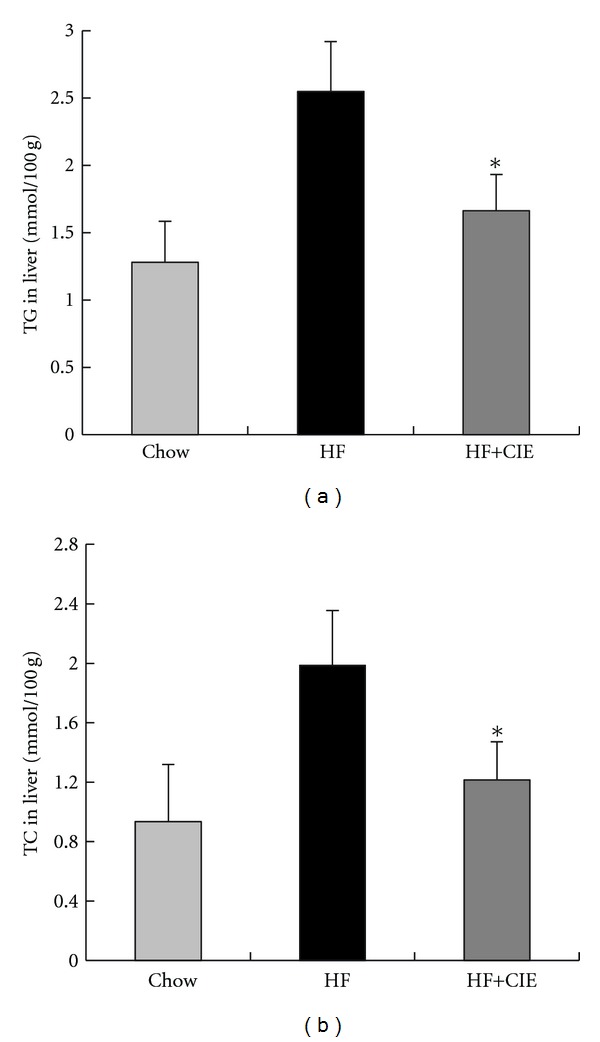
Effects of CIE on hepatic lipid levels in HF diet-fed mice. (a) TG concentrations in liver; (b) TC concentrations in liver. Values are expressed as means ± S.E. (*n* = 7; *P < 0.05, versus the HF group).

The liver, one of the insulin-sensitive tissues, plays a pivotal role in the processes of hyperglycemia and dyslipidemia [38, 39]. Therefore, we examined the lipid contents in the liver of the mice. As shown in Figure 4, the levels of TG and TC in liver of HF group were increased 2.1 and 2.2 times, respectively, compared to the Chow group (*P* < 0.05). Supplementation with CIE significantly reduced TG and TC accumulation in liver compared to the HF group (*P* < 0.05). This finding suggests that CIE could block the HF diet-induced lipid accumulation in the liver. 

### 3.5. CIE Inhibits the Transactivities of PPAR*γ*, LXR*α*, and LXR*β*

**Figure 5:**
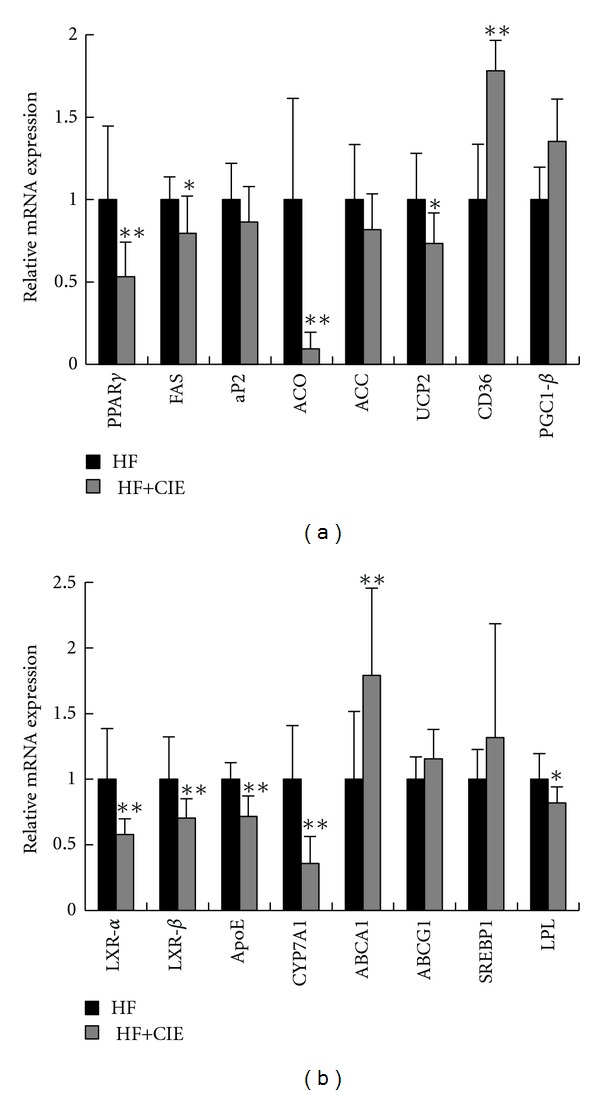
Effects of CIE on the relative mRNA expression in liver tissue. (a) PPAR*γ* and its target genes. (b) LXR and its target genes. Beta-actin was used as an internal control. Values are expressed as means ± S.E. (*n* = 7; *P < 0.05, **P < 0.01, versus the HF group).

To determine the mechanism of CIE ameliorates disorders of glucose and lipid metabolism, related genes' expressions in the liver were examined, as shown in Figures 5(a) and 5(b). PPAR*γ*, one of the most important nuclear receptor transcription factors, regulates the expression of a group of genes involved in lipid and glucose metabolism. Compared with the HF group, CIE treatment notably decreased the mRNA expression of PPAR*γ* in the mouse liver. Moreover, the mRNA levels of PPAR*γ* target genes, including fatty acid synthase (FAS), acyl-CoA oxidase (ACO), and uncoupling protein 2 (UCP2), were also significantly decreased in the livers of HF+CIE group compared with the HF group. However, the expression of adipose fatty acid-binding protein (aP2), acetyl-CoA carboxylase (ACC), and PPAR coactivator1-*β* (PGC1*β*) were not changed significantly by CIE treatment. CIE increased the levels of CD36 mRNA, which is involved in low-density lipoprotein oxidation. These results indicate that CIE plays a role in the regulation of lipid and glucose homeostasis by regulating the expression of PPAR*γ* and its target genes.

Next, we analyzed the mRNA abundance of LXR and its target genes that regulate fatty acids, cholesterol synthesis, and glucose metabolism, such as apolipoprotein E (ApoE), cytochrome P450 7A1 (CYP7A1), lipoprotein lipase (LPL), ATP-binding cassette subfamily G member 1 (ABCG1), ATP-binding cassette transporter A1 (ABCA1), and sterol regulatory element-binding transcription factor 1 (SREBP1), in the liver tissue of CIE-treated and untreated mice. As shown in Figure 5(b), the expression levels of ApoE, CYP7A1, LPL, LXR*α*, and LXR*β* were significantly decreased in CIE-treated mice. There was no significant difference in the mRNA expression levels of ABCG1 and SREBP1 in the liver of the HF group compared with the HF+CIE group. These results indicate that CIE plays a role in metabolic disorders partly through LXR signaling.

## 4. Discussion

Citrus peels are rich in flavonoids that have various biological activities. We found that the major flavonoids in CIE were naringin, hesperidin, and poncirin, of which the highest was naringin. This is different from the findings from *Citrus unshiu* peel extract, which showed that the flavonoid compositions were hesperidin, narirutin, and naringin, with hesperidin being the highest [18]. Both hesperidin and naringin exhibit various biological and pharmacological effects, including antitumor, antiinflammatory, and antioxidant activities, and the potential to improve hyperglycemia, dyslipidemia, and hepatic steatosis in Type 2 diabetes [21, 40–42]. Hesperidin also exhibited hypoglycemic activity in STZ-induced diabetic rats [43]. In a clinical trial, it was demonstrated in hypercholesterolemia patients that naringin supplementation reduces LDL-c by 17% and TC by 14% [7]. Yoon et al. suggested that poncirin promotes osteoblast differentiation and prevents adipogenesis in mesenchymal stem cells [44]. Our results suggest that CIE may be used in the prevention and treatment of the metabolic disorders.

Our animal study showed that CIE could lower blood glucose levels and improve glucose tolerance. In the HF group, body weight gain, serum TC and LDL-c levels, and liver TG and TC levels were significantly increased relative to those in the Chow group and were improved by CIE supplementation. Similar to *Citrus unshiu* peel extract, the CIE also significantly reduced hepatic lipid content as well as blood glucose level. Furthermore, dietary intake of CIE effectively reduced body weight gain and epididymal WAT size in the experimental mice. These positive effects were due to the citrus flavanones such as naringin and hesperidin. We noticed that the food intake was not changed between the groups of HF and HF mixed with CIE, suggesting that the weight-reducing effects of CIE are not caused by suppressing appetite. Also, the fecal lipids were not altered in the CIE-treated mice, suggesting that the body weight reducing effect of CIE is not caused by the inhibition of lipid absorption in the intestine. These results provide convincing evidence that the extract of *Citrus ichangensis *has prevented HF diet-induced obesity and related metabolic disorders.

*Citrus aurantium* flavonoids show antiadipogenesis activity by downregulating the expression of PPAR*γ* in 3T3-L1 cells. In the present study, CIE treatment decreased the gene expression of major glucose and lipid metabolism regulators, including PPAR*γ* and LXRs. The active element of *Citrus ichangensis* probably is naringin, that is, the richest flavonoid in the extract. PPAR*γ* is a major nuclear receptor transcription factor for adipogenesis and lipogenesis. It regulates the expression of a group of genes, including CD36, ACC, ACO, UCP2, FAS, and aP2, which are related to fatty acid synthesis, oxidation, and adipogenesis. It has been shown that PPAR*γ* antagonists can prevent and treat HF diet-induced obesity [45]. Gong et al. reported that suppressing PPAR*γ*-activity can inhibit adipocyte differentiation *in vitro* [46]. Our *in vivo* studies have showed that CIE efficiently suppressed the gene expression of PPAR*γ* in the liver tissue. The expressions of FAS, aP2, ACO, and UCP2 were also significantly decreased in the liver of mice treated with CIE. Moreover, the lipid levels of the liver were significantly lower in the HF+CIE group than in the HF group. These results indicate that CIE may reduce fat weight through the regulation of PPAR*γ* signaling.

To detect other prospective molecular targets through which CIE exhibits anti-metabolic disorders effects, we examined the transactivity of LXR in liver tissue. LXR is known to play a major role in regulation of cholesterol, fatty acid, and glucose homeostasis metabolism [47]. It has been demonstrated that naringenin may decrease serum lipid levels by inhibiting the activation of LXR*α* [26], indicating that the inhibition of LXR also has a therapeutic role. In this study, both LXR*α* and LXR*β* transactivities were inhibited by CIE. The mRNA expression of LXR target genes such as ApoE, CYP7A1, ABCA1, ABCG1, SREBP1, and LPL were further confirmed. The expression levels of ApoE, CYP7A1, and LPL were significantly decreased in the CIE+HF group in comparison to the HF group. However, ABCG1 mRNA expression was not changed significantly by CIF treatment. These results demonstrate that CIE regulates cholesterol and glucose metabolism partly through LXR antagonism in high-fat diet-induced obese mice.

In conclusion, we found that CIE prevents the development of obesity induced by a HF diet and lowers hyperlipidemia and hyperglycemia, while protecting against the lipid accumulation in the liver. These effects may involve multi-molecular targets, including the inhibition of PPAR*γ* and LXRs, in the liver tissue. Our results suggest that *Citrus ichangensis* could be used as a dietary supplement for antiobesity and hyperlipidemia-lowering therapy. However, the mechanism needs to be further investigated.

## Figures and Tables

**Table 1:** Sequences of the primers used in real-time PCR.

Gene	Forward primer	Reverse primer
*β*-Actin	TGTCCACCTTCCAGCAGATGT	AGCTCAGTAACAGTCCGCCTAGA
LXR*α*	GAGTGTCGACTTCGCAAATGC	CCTCTTCTTGCCGCTTCAGT
LXR*β*	CAGGCTTGCAGGTGGAATTC	ATGGCGATAAGCAAGGCATACT
ABCA1	GGCAATGAGTGTGCCAGAGTTA	TAGTCACATGTGGCACCGTTTT
ABCG1	TCCCCACCTGTAAGTAATTGCA	TCGGACCCTTATCATTCTCTACAGA
ApoE	GAACCGCTTCTGGGATTACCT	TCAGTGCCGTCAGTTCTTGTG
CYP7A1	GTGGTAGTGAGCTGTTGCATATGG	CACAGCCCAGGTATGGAATCA
SREBP1	GGCTATTCCGTGAACATCTCCTA	ATCCAAGGGCATCTGAGAACTC
LPL	ATCGGAGAACTGCTCATGATGA	CGGATCCTCTCGATGACGAA
PGC-1*β*	GGGTGCGCCTCCAAGTG	TCTACAGACAGAAGATGTTATGTGAACAC
PPAR*γ*	CGCTGATGCACTGCCTATGA	AGAGGTCCACAGAGCTGATTCC
aP2	CATGGCCAAGCCCAACAT	CGCCCAGTTTGAAGGAAATC
ACC	GAATCTCCTGGTGACAATGCTTATT	GGTCTTGCTGAGTTGGGTTAGCT
ACO	CAGCACTGGTCTCCGTCATG	CTCCGGACTACCATCCAAGATG
UCP-2	GGGCACTGCAAGCATGTGTA	TCAGATTCCTGGGCAAGTCACT
CD36	GCTTGCAACTGTCAGCACAT	GCCTTGCTGTAGCCAAGAAC
FAS	CTGAGATCCCAGCACTTCTTGA	GCCTCCGAAGCCAAATGAG
